# Penetrating Wound of the Orbit with Dislodgement of the Vomer

**Published:** 1912-01

**Authors:** 


### Penetrating Wound of the Orbit with Dislodgement of the Vomer

LAST July, while picking fruit, a lad eight years old fell thirty feet. Without injuring the lids, a branch pierced the internal orbital wall, traversed the pharynx and impinged upon the opposite wall of that cavity. In its passage through the pharynx it dislodged a large portion of the vomer which was expectorated a couple of days later.

The lad's misfortune lies in the fact that the cornea suffered an abrasion which became infected from the pus that soon bathed the eye, and resulted in a perforating ulcer with a subsequent staphyloma. As is not unusual the intra-ocular tension was raised which necessitated an iridectomy. Up to the present there is useful vision.

The feature of the case which must be rare if not unique is the dislodgement of the vomer.

The haemorrhage following the accident was severe and Dr. Pearson, to whom I am indebted for the case, had considerable difficulty in controlling it.

510 Franklin St.

